# The Design of a Multistage Monitoring Protocol for Dendritic Cell-Derived Exosome (DEX) Immunotherapy: A Conceptual Framework for Molecular Quality Control and Immune Profiling

**DOI:** 10.3390/ijms26125444

**Published:** 2025-06-06

**Authors:** Ramón Gutiérrez-Sandoval, Francisco Gutiérrez-Castro, Natalia Muñoz-Godoy, Ider Rivadeneira, Adolay Sobarzo, Luis Alarcón, Wilson Dorado, Andy Lagos, Diego Montenegro, Ignacio Muñoz, Rodrigo Aguilera, Jordan Iturra, Francisco Krakowiak, Cristián Peña-Vargas, Andrés Toledo

**Affiliations:** 1 Department of Oncopathology, OGRD Alliance, Lewes, DE 19958, USA; consultorusa@biogenica.org (C.P.-V.); ops@ogrdalliance.org (A.T.); 2 Department of Cancer Research, Flowinmunocell-Bioexocell Group, 08028 Barcelona, Spain; servicios@flowinmunocell.cl (F.G.-C.); contacto@flowinmunocell.cl (N.M.-G.); 3 Department of Outreach and Engagement Programs for OGRD Consortium, Charlestown KN0802, Saint Kitts and Nevis; iderlautaro@gmail.com (I.R.); luisantonioalarconcofre@gmail.com (L.A.); wdoradoortega@gmail.com (W.D.); lagosandy@gmail.com (A.L.); dn.montenegro.c@gmail.com (D.M.); kinesiologo@recell.cl (I.M.); rodrigo1982aguilera@gmail.com (R.A.); jiconsultant@ogrdconsorcio.com (J.I.); 4 Departamento de Ciencias Biológicas y Químicas, Facultad de Ciencias, Universidad San Sebastián, Lientur 1457, Concepción 4080871, Chile; adolay.sobarzo@uss.cl; 5 Department of Molecular Oncopathology, Bioclas, Concepción 4030000, Chile; tecnologo@bioclas.cl

**Keywords:** structured immunomonitoring, STIP, dendritic cell-derived exosomes, phenotypic immune classification, cytokine logic ratios, non-pharmacodynamic platform, ex vivo immune profiling, vesicle-based quality control

## Abstract

The increasing complexity of dendritic cell (DC)-derived exosome (DEX) immunotherapy demands structured monitoring protocols capable of translating molecular activity into actionable clinical outputs. This study proposes a standardized, multistage immunomonitoring framework designed to evaluate immune activation, cytokine polarization, and product integrity in DEX-based therapies. The protocol integrates open access methodologies—flow cytometry, cytometric bead array (CBA), and Western blotting—to assess CD69/CD25 activation, Th1/Th2/Th17 cytokine profiles, and vesicle identity across distinct checkpoints. These outputs are consolidated within the Structured Immunophenotypic Traceability Platform (STIP), which applies logic-based classifications (Type I–III) to support reproducible stratification of immune responses. Functional validation was performed through ex vivo co-culture models, enabling real-time interpretation of immune polarization, cytotoxic potential, and batch consistency. These outputs are supported by previous experimental validations published in *Cancers* and *Biomedicines* (2025), where PLPC and DC-derived vesicles demonstrated immunological consistency and a phenotypic stratification capacity. This approach provides a scalable monitoring structure that can support personalized treatment decisions, quality assurance workflows, and integration into regulatory documentation (e.g., CTD Module 5.3) for early-phase, non-pharmacodynamic immunotherapies. This conceptual protocol does not aim to demonstrate therapeutic efficacy but to provide a reproducible documentation framework for real-world immune monitoring and regulatory alignment in vesicle-based immunotherapy.

## 1. Introduction

Cancer remains one of the leading causes of mortality worldwide, with millions of new cases diagnosed each year. While current treatments have demonstrated efficacy in specific contexts, they often present significant limitations [1]. Conventional therapies, such as chemotherapy and radiotherapy, are frequently associated with severe adverse effects and high systemic toxicity, which can compromise patient quality of life and limit long-term therapeutic success [2]. These limitations have catalyzed the pursuit of innovative approaches, with immunotherapy emerging as a prominent alternative due to its capacity to leverage the patient’s own immune system to selectively target malignant cells. Despite major advances in molecular oncology, many treating oncologists still lack access to structured immunomonitoring protocols that translate molecular data into actionable clinical strategies. This gap is particularly evident in the context of exosome-based immunotherapies.

Among immunotherapy modalities, dendritic cell (DC)-based strategies are among the most advanced and promising [3,4]. DCs are key orchestrators of adaptive immunity, capable of processing tumor antigens and presenting them to naïve T lymphocytes, thereby eliciting robust and specific antitumor responses. Pulsing DCs with tumor-specific antigens enable the personalization of treatment according to the molecular signature of each patient’s tumor, increasing selectivity and reducing off-target effects [5,6].

In recent years, there has been growing interest in dendritic cell-derived exosomes (DEXs) as non-cellular immunotherapeutic agents [7]. These extracellular vesicles, enriched with proteins, lipids, and nucleic acids, can amplify immune signaling and mediate intercellular communication across immune compartments. Their physicochemical stability and ability to access immunologically privileged or anatomically challenging tumor niches make them particularly attractive as delivery vectors in cancer immunotherapy [8]. The integration of DEXs with antigen-pulsed DC protocols provides an opportunity to enhance therapeutic reach while preserving specificity and immunogenicity.

Despite these advances, the clinical deployment of DEX-based immunotherapies remains constrained by the absence of standardized monitoring systems capable of capturing dynamic molecular correlates of immune activity. Conventional imaging-based response scales, such as RECIST and iRECIST, remain essential for tumor burden assessment but do not reflect immunological engagement, cytokine polarization, or exosome quality [9,10].

To address this gap, the present work introduces a structured, multistage monitoring protocol specifically designed for DEX immunotherapy. Rather than presenting clinical data, this protocol offers a conceptual framework that integrates accessible laboratory techniques—including flow cytometry, Western blotting, and cytokine profiling using a cytometric bead array (CBA)—to enable molecular-level immune monitoring and batch quality control. All tools referenced are compatible with open access, non-commercial platforms that require no licensing, registration, or proprietary software, ensuring reproducibility and scalability in both academic and translational settings.

This methodological proposal does not intend to validate clinical efficacy but to provide a reproducible and adaptable platform that supports the next generation of DEX-based immunotherapy trials. Future studies will focus on the application and prospective validation of this protocol across diverse patient cohorts and clinical scenarios.

Beyond its methodological scope, this protocol serves as a clinical decision-support tool for non-molecular oncologists. It is designed to translate ex vivo immune monitoring results—typically encoded in flow cytometry plots, Western blot bands, or cytokine heatmaps—into logic-governed classifications that are operationally actionable. Rather than introducing new techniques, the protocol integrates well-established, open access tools—such as CBA kits, Western blotting, and standard confluence analysis—into a reproducible framework suited for translational deployment.

In this context, we introduce the Structured Immunophenotypic Traceability Platform (STIP), a modular classification system that consolidates kinetic metrics (e.g., Δ confluence), cytokine polarization (e.g., IFN-γ/IL-10), and non-lethal divergence signals into structured technical dossiers. The framework does not aim to establish therapeutic efficacy, but rather phenotypic compatibility and traceability across vesicle batches and cell types. This framework was conceptually derived from two prior studies that independently validated the immunological consistency and functional stratification potential of PLPC and DC-derived vesicles under experimental conditions [11,12]. The resulting logic tree outputs—categorized into Type I, II, or III immune responses—can be documented, audited, and archived in pre-regulatory formats.

STIP is further designed to support immune stratification in patients who are ineligible for randomized clinical trials or standard pharmacological monitoring, including those receiving experimental vesicle-based therapies with no genomic payload, no systemic toxicity, and no conventional pharmacokinetics.

## 2. Results

**Figure 1:**
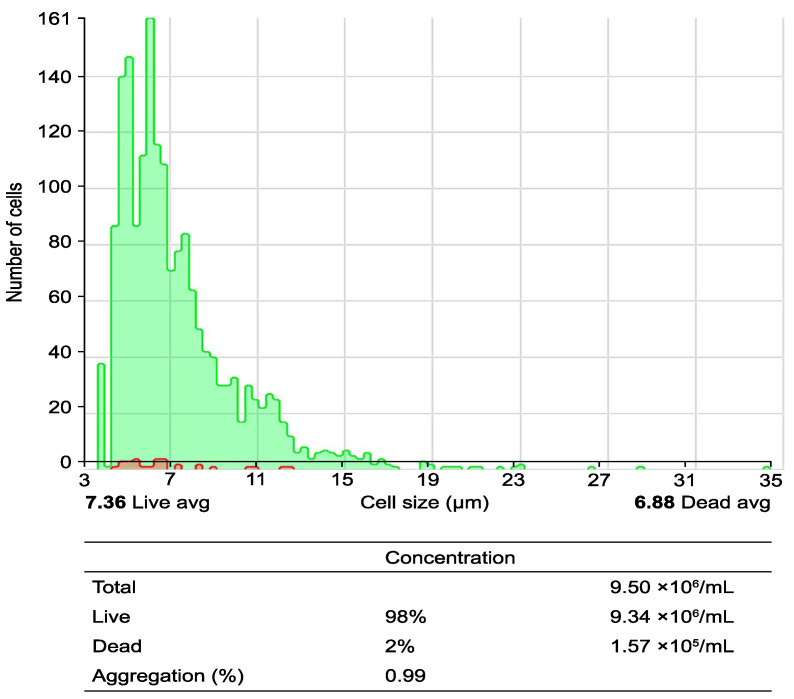
Obtaining peripheral blood mononuclear cells (PBMCs) from peripheral blood using a Ficoll–Hypaque gradient: the process of obtaining PBMCs through a density gradient via centrifugation is illustrated. The upper histogram illustrates the cell size and cell count per analyzed field, differentiating between live cells (green area) and dead cells (red area). The lower table corresponds to the quantification of live and dead cells via analysis with the Countess 3 Automated Cell Counter (number of PBMCs per mL before the seeding process).

Monitoring in DC immunotherapy is crucial to ensure the efficacy of treatment in cancer patients. The first key step in this type of DC immunotherapy is the isolation of progenitor cells, specifically peripheral blood mononuclear cells (PBMCs). Separation is carried out using density gradients, which enables an enriched fraction of monocytes, lymphocytes, and other hematopoietic cells to be obtained, which are essential for subsequent differentiation into DCs [11] (Figure 1). The viability of the progenitor cells is crucial because any contamination would compromise the quality of the resulting DCs.

**Figure 2:**
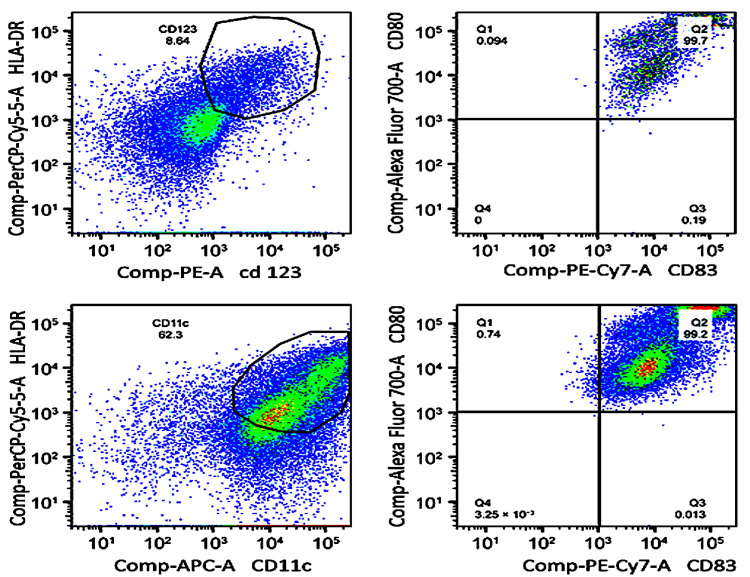
Flow cytometry-based monitoring of dendritic cell (DC) maturation. Dot plots show the phenotypic distribution of plasmacytoid (top row) and myeloid (bottom row) DC subpopulations after in vitro differentiation. Top left: CD123⁺/HLA-DR⁺ cells; top right: CD80⁺/CD83⁺ phenotype. Bottom left: CD11c⁺/HLA-DR⁺ cells; bottom right: CD80⁺/CD83⁺ phenotype. All markers were detected by fluorochrome-conjugated monoclonal antibodies.

Following the isolation of PBMCs, they are differentiated and matured. The differentiation of monocytes into DCs is induced using specific cytokines such as GM-CSF and IL-4, and this process is continuously monitored to ensure that the cells reach the appropriate immature state. Maturation is achieved by the addition of proinflammatory cytokines such as TNF-α and IL-1β, allowing DCs to efficiently present tumor antigens **(**Figure 2**).** Monitoring is essential to ensure that the DCs can fulfill their key immunological function and trigger a specific antitumor response.

One of the most critical parameters to control in this process is cell viability. Viability must be maintained above 90% to ensure that the DCs can fulfill their function of activating T lymphocytes and orchestrating a specific immune response against cancer. For this purpose, techniques such as flow cytometry and cell exclusion assays, such as the Trypan blue exclusion test, were used, which allow the proportion of viable cells to be measured to ensure that the culture maintains its integrity throughout the process [13,14]. The parameters that were evaluated and their expected values are presented in Table 1.

CD25 corresponds to the α-chain of the interleukin-2 (IL-2) receptor. Cytokine levels were quantified using the cytometric bead array (CBA), an open access flow cytometry-based platform. All values reflect expected ranges based on validated co-culture assays using pulsed DCs and autologous T cells.

Furthermore, at this initial stage, the quality control of the exosomes is essential because these extracellular vesicles play a crucial role in the amplification of the immune response. The exosomes’ size and concentration are measured to ensure that they meet the parameters required to function as carriers of tumor antigens. Analytical techniques such as Nanosight have proven effective for this evaluation [15].

### 2.1. Optimization and Characterization in the Molecular Laboratory

The monitoring and optimization of dendritic cell (DC) immunotherapy require a combination of clinical and molecular assessments. While basic monitoring focuses on evaluating the biological quality of DCs and exosomes, optimization at the molecular laboratory level is centered on refining technical variables to ensure the consistency, reproducibility, and effectiveness of the treatment in cancer patients. This includes the implementation of standardized workflows for cell separation, differentiation, vesicle isolation, and immune functionality testing, all designed to minimize batch-to-batch variability and enhance translational scalability.

#### 2.1.1. Optimizing Progenitor Cell Isolation and DC Differentiation

Progenitor cell isolation is a critical initial phase that directly influences the differentiation and maturation potential of the resulting DCs. Peripheral blood mononuclear cells (PBMCs) are enriched via density-gradient centrifugation, a method that must be carefully optimized regarding the centrifugal force, gradient composition, and incubation time to preserve cellular integrity and prevent premature activation [16,17]. Higher viability rates not only improve the efficiency of subsequent DC culture steps but also provide a more immunocompetent starting material.

During the DC differentiation and maturation process, the expression of specific markers such as CD80, CD83, and HLA-DR is systematically evaluated using flow cytometry. These markers serve as critical checkpoints to confirm the phenotypic and functional maturation of DCs. Continuous assessment of these markers throughout the culture period enables early corrective actions, ensuring that the final DC population retains the ability to present antigens effectively and stimulate T-cell responses [18].

#### 2.1.2. Structural and Functional Characterization of Exosomes

The characterization of dendritic cell-derived exosomes (DEXs) constitutes a fundamental component in ensuring the functional reliability of the immunotherapy product. Analytical techniques such as nanoparticle tracking analysis (NTA) and Western blotting are used to precisely measure the size distribution, concentration, and surface marker profile of exosomes [19,20]. Parameters such as particle uniformity, membrane integrity, and protein cargo fidelity are essential indicators of exosome quality.

**Figure 3:**
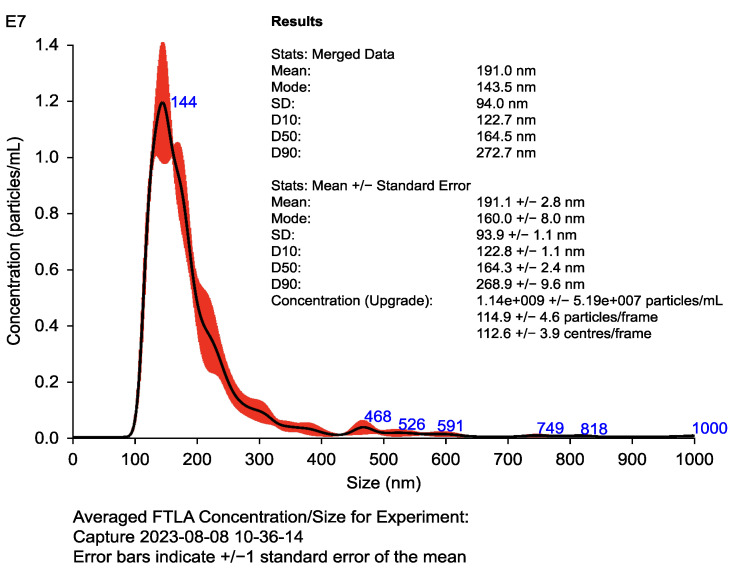
Nanoparticle tracking analysis (NTA) of dendritic cell-derived exosomes (DEXs). Histogram displays particle size distribution (100–150 nm) and concentration (>10^9^ particles/mL). Results confirm exosome uniformity and abundance. Validation was performed via Western blotting using exosomal markers (CD63, CD81, and Alix) and the negative control calnexin, following MISEV 2018 guidelines. Table inset shows mean, mode, and concentration values obtained with Nanosight.

Specifically, the evaluation of markers like CD63, CD81, and Alix confirms the exosomal origin and biogenesis consistency of the vesicles. Additionally, the absence of intracellular contamination is verified by negative controls such as calnexin expression. Together, these assessments guarantee that the exosomes maintain their capacity to function as efficient immunological vehicles capable of delivering bioactive signals within the tumor microenvironment. The full particle distribution and quantitative profile of DEXs are shown in Figure 3, which illustrates the uniformity and concentration range observed via nanoparticle tracking analysis. The results of these quality evaluations are summarized in Table 2.

The parameters listed in Table 2 are commonly used to assess the structural identity, concentration, and purity of dendritic cell-derived exosomes (DEXs). NTA is employed to determine particle size and concentration, while Western blotting is used to confirm the expression of exosomal and control markers. Calnexin serves as a negative control to verify the absence of contaminating intracellular material. All evaluations are based on standardized open access methodologies and aligned with current recommendations from MISEV guidelines.

#### 2.1.3. Advanced Quality and Functionality Assessment

In addition to structural properties, it is crucial to assess the functional capacity of exosomes. This includes the induction of T-cell activation and the production of key cytokines such as IFN-γ, which is measured using the cytometric bead array (CBA) [21]. This functional assessment ensures that exosomes fulfill their role as amplifiers of the antitumor immune response.

To obtain high-purity progenitor cells, we specifically adjusted the cell separation protocol by fine-tuning parameters such as centrifugation speed, separation medium density, and incubation temperature. These adjustments were tailored and validated during the course of our study to address specific challenges observed in our experimental setup, significantly increasing the viability of the isolated PBMCs and enhancing the efficiency of subsequent differentiation. While these modifications build upon established protocols, their integration into a cohesive workflow is a contribution unique to this work, ensuring reproducibility and adaptability for similar laboratory applications.

This manuscript does not report original experimental data (e.g., raw Western blot images, flow cytometry plots, or cytokine curves) because its objective is to propose a structured monitoring protocol—not to present clinical or in vitro results. All values shown in tables and figures reflect expected reference ranges derived from previously validated protocols and serve as structural placeholders within the conceptual framework presented here. The protocol integrates open access methods suitable for reproducible, license-free application, and is intended for future clinical and translational implementation.

#### 2.1.4. Immune Monitoring Protocol

Immune monitoring in pulsed dendritic cell (DC) and exosome immunotherapy is crucial to fine-tune and optimize the immune system’s response to treatment. This approach focuses on accurately measuring T-cell activation; characterizing the Th1, Th2, and Th17 immune profiles; and assessing apoptosis in tumor cells [22]. T-cell activation, specifically of the CD4+ and CD8+ subtypes, is considered a critical indicator, as these cells are responsible for orchestrating the antitumor response by destroying malignant cells.

Flow cytometry is a key tool used to assess the expression of activation markers such as CD69 and CD25. These markers provide a direct measure of the level of cellular activation among T cells [23]. The cytometric bead array (CBA) enables the analysis of the production of key cytokines, such as IFN-γ and IL-12, which are essential for an effective immune response. The robustness of T-cell activation is measured by the proportion of activated CD4+ and CD8+ cells, which is expected to be higher than 70% after co-culture with pulsed DCs [24].

**Figure 4:**
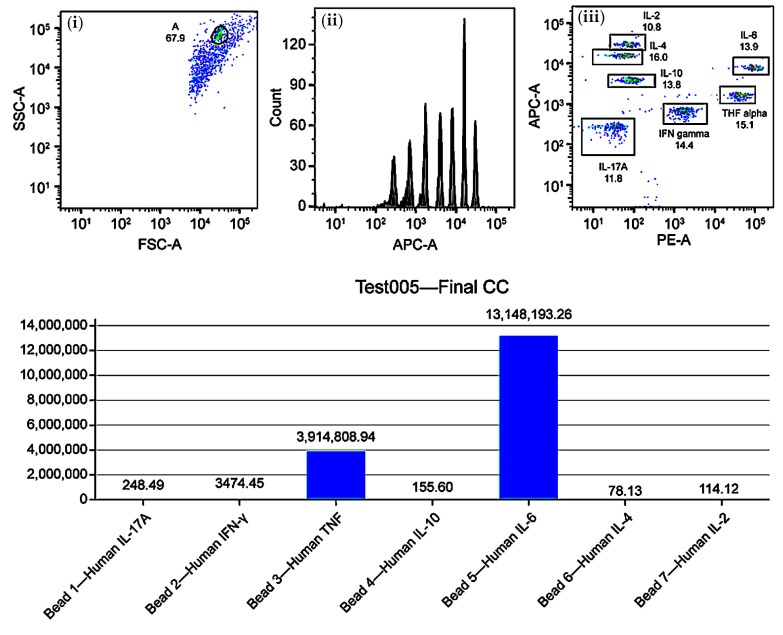
Immune profiling of Th1, Th2, and Th17 cytokines in mature dendritic cell (DC) cultures using cytometric bead array (CBA). Upper panels: (i) FSC-A vs. SSC-A dot plot for event gating, (ii) APC-A histogram showing fluorescence intensity, (iii) APC-A vs. PE dot plot for cytokine clustering. Lower panel: bar graph of normalized cytokine concentrations (pg/mL) for IL-17A, IFN-γ, TNF-α, IL-10, IL-6, IL-4, and IL-2. Results indicate a Th1-skewed profile with predominant IL-6 and TNF-α expression.

The immunological characterization of dendritic cell cultures was performed using flow cytometry and cytometric bead array (CBA), enabling quantification of Th1-, Th2-, and Th17-related cytokines. As shown in Figure 4, the CBA data revealed a Th1-skewed cytokine profile, with predominant expression of IFN-γ, IL-6, and TNF-α, consistent with a cytotoxic immune orientation favorable for antitumor responses.

Furthermore, the analysis of Th1, Th2, and Th17 immune profiles is crucial to characterize the polarization of the immune response. A Th1 profile is ideal for a cytotoxic response against tumor cells, mediated by cytokines such as IFN-γ and IL-12. In contrast, an elevated Th2 profile, associated with cytokines such as IL-4 and IL-10, may have an immunosuppressive effect that compromises therapeutic efficacy [25]. Th17-related cytokines, such as IL-6 and IL-17A, must be carefully monitored to avoid excessive inflammation and immune-mediated toxicity. Flow cytometry and CBA are essential tools for this purpose, enabling dynamic and personalized modulation of the immune protocol [26].

Measuring apoptosis in tumor cells is another key aspect of immunomonitoring. Assays for LDH release, annexin V staining, and caspase activation can confirm the cytotoxic effects of T-cell responses. If suboptimal responses are observed, therapeutic adjustments in exosome dose, DC pulsing, or antigenic load may be warranted [27,28].

### 2.2. Complementary Clinical Follow-Up

It is crucial to monitor exosome immunotherapy to assess treatment effectiveness and make adjustments based on the patient’s response. Such monitoring is mainly based on well-established clinical criteria, such as RECIST and iRECIST, which allow objective changes in tumor lesions to be measured [29]. While RECIST has been widely used to assess the response to conventional therapies, iRECIST has been specifically designed for immunotherapies, addressing unique phenomena such as pseudoprogression, where tumor lesions may temporarily enlarge before showing a reduction due to immune activation [30].

This type of clinical monitoring is complemented by advanced imaging techniques, such as PET-CT with 18F-FDG, which provides a detailed analysis of the metabolic activity of the tumor. The uptake of this radiopharmaceutical by the tumor tissue is directly related to the aggressiveness and cellular metabolism of the cancer [31]. This technique is especially useful in the context of immunotherapy, as it can detect early changes in tumor activity, even before a significant reduction in the size of the lesion occurs, allowing a more accurate assessment of the treatment’s efficacy.

Furthermore, the use of tumor biomarkers offers a molecular window into the patient’s response to treatment. Biomarkers such as carcinoembryonic antigen (CEA), CA-125, and PSA allow indirect measurements of tumor burden and the assessment of disease progression or regression. However, in the context of immunotherapy, these biomarkers may also reflect immune activation. For example, a decrease in PSA levels in prostate cancer patients treated with immunotherapy could indicate a favorable response, while their increase could suggest resistance [32].

Together, clinical criteria, advanced imaging techniques, and tumor biomarkers provide a comprehensive framework that not only assesses the treatment response but also allows for anticipating relapses, adjusting dosages and administration schedules, and identifying patients who would benefit from a change in therapeutic approach [33]. The integration of these elements allows for a dynamic and adaptive approach, maximizing the personalization and effectiveness of treatment in cancer patients.

## 3. Discussion

### 3.1. Impact of Laboratory Results on Treatment Personalization

Laboratory results obtained through molecular monitoring in immunotherapy with DCs and exosomes play a crucial role in the personalization of oncological treatment. The integration of data derived from T lymphocyte activation, cytokine production, and the evaluation of immunological profiles allows the treatment to be adjusted in real time, maximizing its effectiveness and minimizing the incidence of adverse effects. This personalized approach is especially relevant in the context of immunotherapy, where the response of each patient can differ considerably depending on their baseline immune status, tumor burden, and other individual factors [34].

#### 3.1.1. T-Cell Activation and Treatment Adjustments

T-cell activation is a key marker used for assessing the efficacy of immunotherapy, measured through the expression of CD69 and CD25, together with the production of proinflammatory cytokines, such as IFN-γ and IL-2 [35]. These markers provide information on the magnitude of the adaptive immune response, which is essential for combating tumor cells. If T-cell activation levels are suboptimal, adjustments to the therapeutic protocol should be made, such as increasing the dose of pulsed DCs, modifying the antigenic load, or selecting more effective immunological adjuvants [36].

#### 3.1.2. Th1, Th2, and Th17 Immune Profiles: Influence on Response

Th1, Th2, and Th17 immune profiles directly influence the effectiveness of the immune response [37]. A predominant Th1 profile, mediated by cytokines such as IFN-γ and IL-12, is ideal for a cytotoxic response directed against cancer. In cases where a predominance of Th2 or Th17 profiles is detected, which could be related to immunosuppressive or proinflammatory responses, respectively, physicians can adjust the treatment by administering Toll-like receptor (TLR) ligands or interleukin 12 (IL-12), favoring a Th1 response [38,39].

#### 3.1.3. Evaluation of the Quality and Functionality of Exosomes

Exosomes are essential to amplify the immune response. The evaluation of parameters such as the concentration, size, and protein load of exosomes using techniques such as nanoparticle tracking analysis (NTA) and Western blotting ensures their functionality [40,41]. The presence of key exosomal markers, such as CD63 and CD81, is indicative of the quality of these extracellular vesicles [42]. The detection of low efficiency in the activation of T lymphocytes or in the production of cytokines can indicate deficiencies in the quality of exosomes, which would necessitate adjustments in their concentration or improvements in their purification [43].

#### 3.1.4. Adjustments in Immunotherapy Administration

Depending on the molecular results and the patient’s response, the frequency and dose of the immunotherapy can be adjusted. If T-cell activation levels are high, the frequency of administration could be reduced to avoid overstimulation of the immune system and associated adverse effects [44]. Conversely, if the response is insufficient, the frequency or dose of pulsed DCs or exosomes could be increased to enhance immune activation [45].

This dynamic and personalized approach ensures the constant optimization of immunotherapy, adapting to the patient’s progress. The ability to modify the frequency and dose based on patient-specific data differentiates this approach from conventional treatments, offering a considerable advantage in oncological treatment, especially in patients who have not responded to chemotherapy or who are resistant to therapy [44,45].

#### 3.1.5. Clinical Translation: Interpretation Framework for Treating Oncologists

The complexity of immunological data can create significant barriers for oncologists unfamiliar with molecular diagnostics. To address this, we provide a translation framework that connects laboratory findings to clinical decision points. Table 3 summarizes expected values for key immune parameters, their clinical interpretation, and suggested therapeutic actions. This table is designed to be usable in real-time decision making for patient-specific immunotherapy adjustment.

Table 3 provides real-time clinical interpretation of core immunological parameters used in DEX-based monitoring, allowing oncologists to translate immune profiling into concrete therapeutic decisions.

##### Illustrative Clinical Scenarios

Note: These clinical examples are entirely illustrative. They are not derived from real patient data, nor do they imply any therapeutic use, trial, or outcome reported as part of this manuscript. Their sole purpose is to demonstrate potential interpretation pathways within the proposed framework.

Case A: A patient undergoing pulsed DC immunotherapy presents with CD69 at 35%, IFN-γ at 60 pg/mL, and IL-6 within normal limits. This profile indicates insufficient T-cell activation. The clinical team increases the DC pulsing dose and observes CD69 rising to 70% on day 14, confirming effective immune engagement.

Case B: Another patient shows IL-10 levels > 300 pg/mL with a depressed Th1 profile. The treating oncologist interprets this as Th2 dominance with immunosuppressive shift. The protocol is adjusted to reduce exosome frequency and introduce IL-12, resulting in restored Th1/Th2 balance.

### 3.2. Operational Requirements and Protocol Scalability

Pulsed dendritic cell-derived exosomes (DEXs) represent a promising strategy in immuno-oncology, offering high modularity, adaptability, and the ability to integrate across a variety of clinical contexts. These vesicle-based therapies can theoretically enhance immune system reprogramming without the risks associated with replicative vectors or genetic engineering platforms. However, the integration of DEX-based immunotherapy into broader applications is influenced by multiple scalability constraints, particularly those related to technical infrastructure, data management, product standardization, and inter-center reproducibility. Central to this challenge is the reliance on advanced molecular monitoring tools—such as flow cytometry, cytometric bead array systems (CBA), and PET-CT imaging—which are essential for evaluating immune responses and tumor dynamics across diverse patient cohorts [46,47].

Flow cytometry provides high-resolution profiling of immune activation markers, lymphocyte subset distributions, and cytokine expression patterns. PET-CT imaging, particularly when using 18F-FDG tracers, enables early metabolic assessment of tumor microenvironmental changes, offering a sensitive biomarker for functional treatment responses [48]. Although these tools are critical for mechanistic validation and translational immunomonitoring, they require specialized instrumentation, standardized acquisition protocols, robust quality assurance programs, and highly trained personnel to ensure consistent data quality across centers—factors that may limit their routine adoption in decentralized or resource-constrained environments [49].

Addressing these limitations will require a strategic focus on system integration, automation, and inter-institutional coordination. Emerging models propose the use of distributed analytical hubs, artificial intelligence-assisted data processing pipelines, and adaptive protocol platforms that allow for the real-time calibration of immune monitoring strategies. The implementation of shared platforms for data acquisition and analysis, coupled with the incorporation of open access, machine learning-enabled workflows, could significantly improve reproducibility and monitoring throughput. Ultimately, the translational success of DEX-based protocols will depend not only on their biological efficacy but also on their operational scalability, flexibility, and standardization within diverse clinical ecosystems [50,51].

#### 3.2.1. Resource Demands and Technical Requirements

The implementation of this protocol requires access to a carefully calibrated set of specialized analytical platforms, including flow cytometers equipped with multiparametric detectors, automated cytometric bead array (CBA) systems for cytokine profiling, and nanoparticle tracking analysis (NTA) devices for exosomal characterization [52]. These instruments are essential for maintaining the necessary levels of analytical sensitivity, specificity, and reproducibility in immunomonitoring workflows. Additionally, technical demands extend beyond instrumentation and include the need for structured sample handling pipelines, validated standard operating procedures (SOPs) for data acquisition, and reproducible frameworks for biomarker quantification and interpretation. All of these elements must rely on personnel with specialized training in immunophenotyping, vesicle biology, and translational data management [53].

To ensure reproducibility and data integrity across immune readouts, all flow cytometry and CBA assays performed under the STIP framework were subject to rigorous quality control standards. Antibody panels and cytokine bead kits were lot-matched and validated under ISO-equivalent QA protocols. Flow cytometers (e.g., BD FACSCanto II) were calibrated daily using CS&T beads and PMT alignment tools.

Biological assays were performed in triplicate, with independent technical replicates for each condition. Replicates were only accepted if the inter-well correlation exceeded R^2^ ≥ 0.95, and cytokine duplicates showed a coefficient of variation (CV%) below 15%. Cytokine quantification curves were accepted only if R^2^ ≥ 0.98. All outputs—CD69, CD25, IFN-γ, and IL-10—were archived in STIP dossiers with full traceability, including operator, lot number, and instrument batch. Each final classification (Type I/II/III) underwent an independent audit by a secondary analyst.

Rather than focusing solely on instrumentation or isolated technical endpoints, the strength of this monitoring protocol lies in its modularity, interoperability, and compatibility with non-commercial, license-free platforms [54]. This architecture enables reproducible assessments across laboratories with heterogeneous infrastructure levels, thus promoting widespread adoption. Importantly, the protocol allows key molecular immune parameters—such as T-cell activation, cytokine polarization, and apoptosis induction—to be integrated into real-time clinical decision-making algorithms for immunotherapy adjustment, dose titration, and progression monitoring, thereby enhancing the precision and adaptability of patient-specific therapeutic strategies [55,56].

A comparative overview of the operational complexity, standardization needs, and functional contributions of key immunomonitoring techniques is provided in Table 4. The purpose is not to evaluate financial metrics but to contextualize the indispensable role of each analytical platform in building a robust and scalable molecular monitoring framework.

This table summarizes the key methodological components involved in immunomonitoring for DEX-based therapies. Flow cytometry enables precise immune profiling, PET-CT imaging supports the early detection of treatment responses through metabolic readouts, and NTA offers a standardized evaluation of the extracellular vesicle size and particle concentration. These tools are essential for molecular-level monitoring and translational reproducibility and have been selected for their compatibility with open access or widely implemented laboratory systems.

#### 3.2.2. Scalability

Protocol scalability is another critical factor to consider. It largely depends on the automation of processes and the implementation of more accessible technologies that enable its adoption in different clinical contexts. These technologies not only simplify workflows but also ensure the consistency and reproducibility of monitoring results across diverse settings. The integration of artificial intelligence tools for data analysis, such as machine learning algorithms and predictive analytics, can further enhance scalability by streamlining data interpretation and enabling real-time adjustments to treatment protocols. This facilitates the adoption of these protocols in hospitals and clinics worldwide, optimizing resources and improving monitoring efficiency [57,58].

Furthermore, the ongoing training of physicians and laboratory technicians in the use of these advanced technologies is crucial to ensure accurate and efficient monitoring, regardless of patient volume. Such training should prioritize hands-on experience and simulations to familiarize staff with both hardware and software components. Developing standardized training programs that include periodic evaluations can help maintain high competency levels, particularly as new technologies emerge [59].

Another strategy to improve scalability is to foster collaboration between research institutions, treatment centers, and biotechnology companies. This collaboration could involve the creation of shared monitoring networks that pool resources and expertise, reducing the duplication of efforts and associated costs. Additionally, implementing standardized protocols across institutions would simplify regulatory compliance and facilitate data sharing for research purposes, accelerating advancements in personalized immunotherapy [60,61].

A detailed analysis comparing the estimated costs associated with automation and institutional collaboration can allow the cost–benefit ratios to be calculated as these protocols are expanded. For example, institutions that invest in shared infrastructure may experience significant reductions in operational expenses over time, enabling them to allocate resources to patient care and further research [62].

Finally, the costs associated with the implementation of this comprehensive monitoring protocol are more than offset by the reduction in hospitalizations and decrease in serious adverse effects that it achieves. Personalized immunotherapy, when combined with efficient monitoring, minimizes treatment-related complications and optimizes outcomes, ultimately supporting broader access, standardized application, and improved outcome predictability across diverse healthcare environments [63].

### 3.3. Limitations and Future Directions

While this protocol is not designed to introduce new laboratory techniques, its contribution lies in its translational configuration and its accessibility for clinicians without molecular specialization. Despite significant advances in immunotherapy with DCs and exosomes, there are several limitations inherent to this therapeutic approach. These limitations are the result of the biological complexity of tumors and the variability in patient responses. Factors such as the total tumor burden, the patient’s baseline immune status, the presence of comorbidities, and the specific genetic profile can significantly influence the effectiveness of the treatment [64,65].

#### 3.3.1. Limitations in the Immune Response

Variability in immune responses poses a significant challenge, influenced by tumor microenvironment heterogeneity and immunosuppressive cell populations. Future strategies must focus on incorporating patient-specific biomarkers and real-time adaptive monitoring to overcome these limitations, which may be related to differences in the composition of the tumor microenvironment, the presence of immunosuppressive cells, and the intrinsic capacity of each individual’s immune system. Furthermore, tumor cell heterogeneity can lead to immune system evasion, hindering the effectiveness of exosomes and DCs [66].

Although the structural and molecular quality control of exosomes—such as size, concentration, and marker expression—provides critical information about batch consistency, it does not directly translate into predictable therapeutic efficacy. This is due to the inherent variability of patient immune profiles, tumor microenvironments, and systemic tolerance thresholds. Even when exosome preparations meet optimal biophysical and biochemical specifications, the clinical outcome can vary significantly depending on individual immunological context. Therefore, any standardization protocol must be interpreted as a prerequisite for safety and reproducibility, but not as a surrogate marker for clinical performance.

To mitigate immunogenic drift across vesicle batches, our monitoring protocol incorporates both structural and functional batch fingerprinting. Structural validation is based on dynamic light scattering (DLS), Fourier-transform infrared spectroscopy (FTIR), and endotoxin quantification, ensuring vesicle uniformity and biochemical purity. In parallel, the STIP framework applies functional evaluation through ex vivo exposure to sentinel immune cell lines, quantifying Δ-confluence, cumulative cytotoxicity signals, and cytokine ratios—particularly IFN-γ/IL-10—to define immune behavior reproducibility [67,68]. Each batch is assigned a Functional Stratification Index (FSI), which enables inter-batch comparability and systematic documentation of immune modulation profiles. This approach has been applied in over 500 vesicle–cell interactions and has demonstrated low inter-batch variability and high phenotypic reproducibility (STIP system patented by OGRD Alliance, 2025; complementary manuscript submitted to a journal within the MDPI group). Collectively, these procedures enable early detection of immune divergence and provide a scalable, regulatory-compliant framework for batch-level traceability in the development of non-pharmacodynamic immunotherapies [69].

#### 3.3.2. Future Directions in Research

Addressing these limitations requires deeper exploration of the mechanisms governing immune responses in cancer patients, particularly those involving tumor microenvironment heterogeneity and immune evasion. Preclinical models and multi-phase clinical trials are essential to identify strategies that optimize T-cell activation and improve the efficacy of DC- and exosome-based therapies. Moreover, the integration of emerging technologies, such as single-cell sequencing and artificial intelligence-driven data analysis, holds significant promise for real-time therapeutic adjustments. Collaborative efforts among research institutions and healthcare providers will be critical in accelerating the implementation of these personalized approaches. The personalization of immunotherapy, considering genetic and molecular factors, will be crucial to improve effectiveness and reduce side effects [70].

Furthermore, the development of emerging technologies, such as gene editing and artificial intelligence, could offer new opportunities to optimize therapies and predict responses in real time. The implementation of advanced monitoring platforms will allow for the continuous adaptation of therapeutic strategies based on individual patient responses [71].

#### 3.3.3. Interdisciplinary Collaborations

Fostering collaboration across disciplines, including molecular biology, oncology, and bioinformatics, will be essential to create a comprehensive approach to addressing the complexities of cancer treatment. Research networks and consortia can facilitate the sharing of data and resources, accelerating the development of new therapies and monitoring strategies [72].

In conclusion, immunotherapy with DCs and exosomes has shown significant potential in the treatment of cancer and will undoubtedly be of great benefit to continued research and in addressing current limitations. A multidisciplinary approach and the integration of new technologies may open up new avenues to increase the effectiveness of these treatments in the future.

These aspects, along with the system’s limitations and forward-looking projections discussed in Section 3.3, highlight the importance of continued refinement and regulatory adaptation of this immunomonitoring framework [73].

## 4. Materials and Methods

To ensure adaptability to different immune and clinical contexts, the STIP system was validated ex vivo using standardized sentinel cell lines representing a wide range of tissue types and oncological phenotypes (e.g., A375, U87-MG, BEWO, and PANC-1). These models mimic variability in immune susceptibility, proliferative kinetics, and cytokine polarization without requiring patient contact. In more than 500 vesicle–cell combinations, STIP has demonstrated reproducibility, divergence consistency, and functional classification capacity (Type I/II/III), supporting its use in translational validation [74].

In addition, the platform includes an adaptive clinical integration model (STIP-RA) that enables the incorporation of patient-level observational data (e.g., functional recovery, imaging, and inflammatory panels) into the logic-tree classification system without requiring molecular profiling or biopsy This extension enables phenotypic documentation in patients excluded from traditional clinical trials while preserving traceability, structural consistency, and regulatory compatibility [75].

A key contribution of this protocol is its ability to incorporate both traditional clinical scales, such as RECIST and iRECIST, and advanced molecular tools, including flow cytometry, cytometric bead array (CBA), and PET-CT imaging. This multidimensional evaluation framework not only deepens the understanding of immune activation and tumor response but also facilitates precise adjustments to treatment regimens. For instance, tracking critical immune parameters such as T-cell activation, cytokine profiles (Th1, Th2, and Th17), and tumor apoptosis markers enables the personalization of therapies to align with the patient’s immune system dynamics. A Th1-dominant profile, characterized by cytokines like IFN-γ and IL-12, supports robust cytotoxic responses, while adjustments can be made for Th2 or Th17 profiles to mitigate immunosuppression or excessive inflammation [76,77].

Table 5 provides a structured overview of the immunomonitoring protocol, detailing operational checkpoints, timing, assays, and expected outputs across each phase of pulsed DEX immunotherapy.

These protocols enable the precise evaluation of crucial therapeutic parameters, including cell viability, T-cell activation, and the production of key cytokines such as IFN-γ and IL-12. Such detailed assessments are indispensable for confirming the efficacy of the therapy and tailoring it to the specific needs of individual patients, as demonstrated in Table 5 [78].

## 5. Conclusions

This work presents a structured, proof-of-concept protocol for integrating molecular and clinical monitoring tools in dendritic cell-derived exosome (DEX) immunotherapy. While it does not claim clinical validation, the protocol offers a reproducible and scalable framework for translational use. It enables real-time treatment adjustment based on immune phenotypes and serves as a methodological reference for future regulatory applications.

The monitoring strategy described here incorporates logic-driven classifications (Type I/II/III) based on kinetic divergence (ΔC, ΔT), cytokine polarization (e.g., IFN-γ/IL-10), and apoptosis-associated biomarkers, producing standardized dossiers for immunotherapeutic documentation. Its design ensures traceability, compatibility with CTD Module 5.3, and adaptability to diverse clinical environments [79].

A key innovation of this protocol lies in its accessibility for non-molecular oncologists, allowing immune monitoring to be conducted without requiring advanced genomic or transcriptomic analysis. As precision oncology expands, the integration of multidimensional immune profiling into everyday practice is critical for tailoring therapy to the patient’s dynamic immune response.

Table 6 summarizes the Th1/Th2/Th17 polarization profiles observed in DEX-based immunomonitoring. Understanding these trends is essential for clinical decision-making, especially in immunotherapy contexts where polarization imbalances can compromise treatment efficacy or trigger adverse events [80].

Beyond its immunological impact, this monitoring approach has profound economic implications. The long-term reduction in hospitalizations, adverse effects, and treatment inefficiencies offsets its implementation cost, which is expected to decrease further with technological advances in automation and AI-assisted analysis [76].

Widespread adoption will depend not only on molecular effectiveness but also on operational scalability, particularly in resource-limited settings. Technologies such as high-throughput cytometry, AI-driven analytics, and digital immuno-mapping tools can streamline workflows and enable decentralized immunomonitoring [78].

Ongoing training of medical teams and collaborative standardization across institutions will be essential to ensure uniform data quality, reproducibility, and protocol adherence [79]. Ultimately, this model supports the alignment of immune dynamics with therapeutic decisions, contributing to the real-world operationalization of personalized immunotherapy [80].

## Figures and Tables

**Table 1:** Functional immune markers for assessing dendritic cell activation and viability.

Marker	Description	Evaluation Method	Expected Value
CD69	Early marker of T-cell activation	Flow cytometry	>70% activated lymphocytes
CD25	Late activation marker; α-chain of IL-2 receptor	Flow cytometry	>60% activated lymphocytes
HLA-DR	Maturation marker for dendritic cells	Flow cytometry	High expression (>80%)
IFN-γ	Key cytokine for Th1 polarization	Cytometric Bead Array (CBA)	100–150 pg/mL
IL-12	Cytokine for induction of Th1 immune profile	CBA	>80 pg/mL

**Table 2:** Key features of dendritic cell-derived exosomes (DEXs) and the analytical methods used for their characterization.

Parameter	Description	Evaluation Method	Optimal Range
Size	Average diameter of exosomes	NTA	100–150 nm
Concentration	Number of exosomes per mL of sample	NTA	>10^9^ particles/mL
CD63	Exosome-specific surface marker	Western blotting	Positive expression
CD81	Extracellular vesicle marker	Western blotting	Positive expression
Alix	Marker of exosome integrity and biogenesis	Western blotting	Positive expression
Calnexin	Negative control for exosomal purity	Western blotting	Not detected

**Table 3:** Clinical interpretation of immune monitoring biomarkers and corresponding therapeutic strategies.

Parameter	Expected Value	Clinical Meaning	Suggested Action
CD69 < 50%	Low T-cell activation	Suboptimal immune engagement	Increase DC dose or adjuvant
IFN-γ < 80 pg/mL	Weak Th1 response	Reduced cytotoxicity	Consider IL-12 co-stimulation
IL-10 > 200 pg/mL	Dominant Th2 suppression	Risk of immune escape	Adjust antigen load or regimen
IL-6 > 500 pg/mL	Inflammatory toxicity	Excessive immune activation	Delay next dose; modulate cytokines

**Table 4:** Core analytical techniques used in DEX-based immunomonitoring.

Monitoring Technique	Operational Complexity	Functional Contribution
Flow cytometry	High	High-resolution quantification of immune activation markers (e.g., CD69, CD25, and HLA-DR)
PET-CT with 18F-FDG	Very high	Sensitive detection of early metabolic tumor responses and treatment-associated dynamics
Nanoparticle Tracking Analysis	Moderate	Characterization of exosome size distribution and concentration (standardized QC metric)

**Table 5:** Stepwise validation protocol for pulsed DEX immunotherapy: checkpoints, assays, and clinical traceability.

No.	Stage	Day	Sample	Exam	Purpose
1	Isolation of PBMCs	Day 1	Peripheral blood	Ficoll density gradient separation	Obtain viable PBMCs suitable for DC differentiation and immunomonitoring workflows
2	Cell viability and integrity	Day 1	Isolated PBMNCs	Trypan Blue or Annexin V assay	Confirm cell viability (>95%) and baseline functional status
3	DC differentiation	Day 7	PBMC culture	Flow cytometry: HLA-DR, CD123, CD11c	Verify phenotypic markers indicating effective differentiation into immature DCs
4	DC maturation	Day 10	Immature DCs	Flow cytometry: CD80, CD83, CD86	Confirm maturation capacity and readiness for lymphocyte co-culture
5	Exosome harvest and QC	Day 12	DC secretome	NTA for size/concentration	Validate exosome yield and structural uniformity (90–120 nm)
6	Immunopotency profiling	Day 12	DC secretome	CBA: Th1, Th2, Th17 cytokines	Evaluate cytokine patterns supporting immune polarization capacity
7	Lymphocyte activation	Day 14	DCs and/or exosomes co-cultured with T cells	Flow cytometry: CD69, CD25	Confirm T-cell activation and effector induction
8	Tumor apoptosis induction	Day 14	Activated T cells + tumor cell co-culture	LDH, caspase activity assays	Measure tumor cell apoptosis as endpoint of immune activation
9	Final product validation	Day 14	Enriched exosome concentrate	Safety, membrane integrity, surface marker validation	Ensure compliance with quality and biosafety standards prior to clinical use

**Table 6:** Immune polarization profiles relevant to DEX-based immunotherapy.

Immune Profile	Key Cytokines	Impact on Therapy
Th1	IFN-γ, IL-12	Promotes robust cytotoxic response; enhances T-cell activation and tumor clearance
Th2	IL-4, IL-10	May suppress cytotoxic responses; potentially reduces therapeutic efficacy
Th17	IL-6, IL-17A	Associated with pro-inflammatory responses; requires modulation to minimize adverse effects

## Data Availability

Raw kinetic data, secretomic profiles, classification parameters, and additional underlying datasets, are available from the corresponding author upon reasonable request. Access to these data may be subject to confidentiality agreements or material transfer conditions related to ongoing regulatory submissions. The full dataset is part of an active corporate editorial pipeline and is managed in accordance with contextual integrity and planned licensing frameworks.

## Abbreviations

APC-A	Allophycocyanin Area
CA-125	Cancer Antigen 125
CBA	Cytometric Bead Array
CD	Cluster of Differentiation
CD80, CD83, CD63, CD81	Cell Differentiation Markers
CTD	Common Technical Document
DC	Dendritic Cell
DEX	Dendritic Cell-Derived Exosome
ΔC	Change in Confluence (Kinetic Divergence Index)
ΔT	Divergence Time (Time to Immunological Shift)
FSC-A	Forward Scatter Area
FSI	Functional Stratification Index
GM-CSF	Granulocyte–Macrophage Colony-Stimulating Factor
HLA-DR	Human Leukocyte Antigen–DR Isotype
IFN-γ	Interferon Gamma
IL-1β	Interleukin 1 Beta
IL-4	Interleukin 4
IL-12	Interleukin 12
iRECISTs	Immune Response Evaluation Criteria in Solid Tumors
LDH	Lactate Dehydrogenase
NTA	Nanoparticle Tracking Analysis
PBMC	Peripheral Blood Mononuclear Cell
PE	Phycoerythrin
PET-CT	Positron Emission Tomography–Computed Tomography
pg/mL	Picograms per Milliliter
PLPC	Phospholipoproteic Complex
PSA	Prostate-Specific Antigen
QC	Quality Control
RA	Real-world Adaptive
RECIST	Response Evaluation Criteria in Solid Tumors
SSC-A	Side Scatter Area
STIP	Structured Immunophenotypic Traceability Platform
Th1, Th2, Th17	T Helper Cell Subtypes
TLR	Toll-Like Receptor
TNF-α	Tumor Necrosis Factor Alpha
